# Biocontrol of Tomato Bacterial Wilt by Foliar Spray Application of a Novel Strain of Endophytic *Bacillus* sp.

**DOI:** 10.1264/jsme2.ME20078

**Published:** 2020-10-02

**Authors:** Hui-Zhen Fu, Malek Marian, Takuo Enomoto, Ayaka Hieno, Hidemasa Ina, Haruhisa Suga, Masafumi Shimizu

**Affiliations:** 1 The United Graduate School of Agricultural Science, Gifu University, 1–1 Yanagido, Gifu, Gifu 501–1193, Japan; 2 Faculty of Applied Biological Sciences, Gifu University, 1–1 Yanagido, Gifu, Gifu 501–1193, Japan; 3 College of Agriculture, Ibaraki University, 3–21–1 Chuuo, Ami, Inashiki, Ibaraki 300–0393, Japan; 4 Life Science Research Center, Gifu University, 1–1 Yanagido, Gifu, Gifu 501–1193, Japan

**Keywords:** endophytic *Bacillus*, foliar spray, biological control, induced systemic resistance, *Ralstonia pseudosolanacearum*

## Abstract

The aim of the present study was to identify a strain of endophytic *Bacillus* species that control tomato bacterial wilt by foliar spray application. Fifty heat-tolerant endophytic bacteria were isolated from the surface-sterilized foliar tissues of symptomless tomato plants that had been pre-inoculated with the pathogen *Ralstonia pseudosolanacearum*. In the primary screening, we assessed the suppressive effects of a shoot-dipping treatment with bacterial strains against bacterial wilt on tomato seedlings grown on peat pellets. *Bacillus* sp. strains G1S3 and G4L1 significantly suppressed the incidence of tomato bacterial wilt. In subsequent pot experiments, the biocontrol efficacy of foliar spray application was examined under glasshouse conditions. G4L1 displayed consistent and significant disease suppression, and, thus, was selected as a biocontrol candidate. Moreover, the pathogen population in the stem of G4L1-treated plants was markedly smaller than that in control plants. A quantitative real-time PCR analysis revealed that the foliar spraying of tomato plants with G4L1 up-regulated the expression of *PR-1a* and *LoxD* in stem and *GluB* in roots upon the pathogen inoculation, implying that the induction of salicylic acid-, jasmonic acid-, and ethylene-dependent defenses was involved in the protective effects of this strain. In the re-isolation experiment, G4L1 efficiently colonized foliar tissues for at least 4‍ ‍weeks after spray application. Collectively, the present results indicate that G4L1 is a promising biocontrol agent for tomato bacterial wilt. Furthermore, to the best of our knowledge, this is the first study to report the biocontrol of bacterial wilt by the foliar spraying with an endophytic *Bacillus* species.

Tomato (*Solanum lycopersicum* L.) is one of the most consumed and economically important vegetables worldwide, second only to potato (*Solanum tuberosum* L.). According to data provided by FAOSTAT, currently estimated global tomato production is approximately 182 million tons (FAOSTAT, 2018). Bacterial wilt, caused by *Ralstonia solanacearum* and *Ralstonia pseudosolanacearum*, is one of the most destructive diseases of tomato, and leads to tremendous economic losses (Elphinstone, 2005; Artal *et al.*, 2012). The disease is more prevalent in tropical, subtropical, and warmer temperate regions of the world; however, epidemics have been rapidly expanding toward colder temperature regions at high latitudes and altitudes because of the emergence of cold-tolerant strains of the pathogen, as well as global warming (Jiang *et al.*, 2017; Wang *et al.*, 2018). The most common and widespread method for controlling bacterial wilt is soil disinfestation using chemical fumigants (Yuliar *et al.*, 2015). However, soil fumigation has often failed to sufficiently suppress bacterial wilt (Suchoff *et al.*, 2019). Moreover, chemical fumigants are highly toxic to a wide variety of organisms, including humans; thus, the use of these chemicals may exert a serious negative effect on human health and the environment. The cultivation of resistant cultivars or susceptible cultivars grafted onto resistant rootstocks is another conventional approach to control bacterial wilt (Yuliar *et al.*, 2015). The use of resistant cultivars and resistant rootstocks is considered the most effective and eco-friendly method for the management of bacterial wilt. However, the disease is often reported in both commercial resistant cultivars and grafted tomatoes (Kim *et al.*, 2016; Rossato *et al.*, 2018). Therefore, there is an urgent need to develop alternative or supplementary control measures against tomato bacterial wilt, and biocontrol using antagonistic microorganisms has recently attracted increasing attention.

Many researchers have extensively examined the development of biocontrol methods for bacterial wilt. Since bacterial wilt is a soil-borne disease, the majority of studies have focused on the use of antagonistic microorganisms dwelling in root-associated zones, including the rhizosphere and endosphere (inside roots), for the protection of plant roots from pathogen attack (Eljounaidi *et al.*, 2016; Mamphogoro *et al.*, 2020). Accordingly, a variety of antagonistic microorganisms, such as *Pseudomonas* spp., *Bacillus* spp., *Streptomyces* spp., non-pathogenic *Ralstonia* spp., and* Trichoderma* spp., have recently been identified from the rhizospheres or interior of roots as effective biocontrol agents against bacterial wilt (Chandrasekaran *et al.*, 2016; Farahat *et al.*, 2016; Cao *et al.*, 2018; Marian *et al.*, 2018; Yendyo *et al.*, 2017; Ling *et al.*, 2020). It is widely accepted that root colonization by introduced biocontrol agents (BCAs) is an essential prerequisite for the successful biocontrol of soil-borne diseases (Sachdev and Singh, 2018). In the majority of previous studies on the biocontrol of bacterial wilt, selected BCAs were applied to roots by soil drenching or root dipping before pathogen inoculation or the transplantation of seedlings into infected soil. However, their biocontrol performance in the field has often been short-lived, most likely because of a decline in the introduced BCA populations due to the influence of abiotic and biotic factors. Since bacterial wilt occurs continuously during warm weather seasons, it is important to maintain a sufficient population of BCAs in order to protect plants during the warmer months, from early summer to early fall. Therefore, many researchers have repeated drench applications of BCAs to roots at regular intervals after transplantation to ensure consistent colonization (Nguyen and Ranamukhaarachchi, 2010; Ramesh and Phadke, 2012; Yendyo *et al.*, 2017). However, the “booster” drench application of a sufficient volume of a BCA inoculum to protect the entirety of the roots is technically difficult, labor-intensive, and costly.

Some endophytic and rhizospheric microorganisms activate systemic disease resistance, which is generally called induced systemic resistance (ISR), to protect host plants from a wide variety of pathogens. To date, many studies have reported the successful control of both soil-borne and air-borne diseases by inoculating plant roots with ISR-inducing BCAs (Iavicoli *et al.*, 2003; Hase *et al.*, 2008; Hyakumachi *et al.*, 2013; Martínez-Hidalgo *et al.*, 2015; Yamamoto *et al.*, 2015; Li *et al.*, 2017; Molinari and Leonetti, 2019). In contrast, the foliar application of ISR-inducing BCAs has seldom been used for the biocontrol of soil-borne diseases. It is theoretically possible to control soil-borne pathogens via the induction of systemic resistance from the foliar application of resistance-inducing agents. The foliar spraying of validamycin A, an antibiotic, may induce systemic resistance against soil-borne diseases in plants; thus, this antibiotic has been registered for the control of eggplant diseases, including bacterial wilt, in Japan (Ishikawa *et al.*, 2007). Based on these findings, we assumed that the foliar application of ISR-inducing endophytic BCAs will provide a new practical approach for controlling bacterial wilt. The booster application of BCAs by foliar spraying will likely be easier, less labor-intensive, and less costly than soil-drenching. In foliar applications, it is desirable to use microorganisms that survive under adverse environmental conditions and efficiently colonize foliar tissues because the introduced BCAs are exposed to various abiotic stresses, such as drought and UV radiation, after spray application (Marian and Shimizu, 2019). Consequently, we selected endophytic *Bacillus* species as the most suitable candidate for a BCA. *Bacillus* species display high resilience to diverse environmental stresses due to their endospore-producing ability (Nicholson, 2002; Shafi *et al.*, 2017). Thus, they are one of the most studied BCAs against foliar disease (Alippi *et al.*, 2000; Collins and Jacobsen, 2003; Ji *et al.*, 2013; Li *et al.*, 2015; Zhang *et al.*, 2017). The advantage of using endophytic *Bacillus* is that once it has colonized the interior of the plant tissue, it is protected from environmental stresses and fluctuations.

The goal of the present study was to select a strain of endophytic *Bacillus* that effectively suppresses tomato bacterial wilt by foliar spray application. We isolated heat-tolerant bacteria from the surface-sterilized foliar tissues of tomato plants and screened them for their ability to induce bacterial wilt resistance in tomato plants.

## Materials and Methods

### Plants

A susceptible tomato cultivar, Ponderosa, was used throughout this study. The seeds were surface sterilized in 70% (v/v) ethanol for 1‍ ‍min and 2% (v/v) sodium hypochlorite for 5‍ ‍min, and then rinsed six times with sterile distilled water (SDW). Sterilized seeds were germinated at 25°C in the dark for 3 days on filter paper moistened with SDW. Seedlings used for bacterial isolation and the primary screening experiment were prepared by growing the germinated seeds on peat pellets (Jiffy-7 pellets; Jiffy Products International AS) in a glasshouse maintained at 28–30°C under natural sunlight (one seed per pellet for bacterial isolation and five seeds per peat pellet for the primary screening experiment). In pot experiments, germinated seeds were sown in plastic trays (Bee pot Y-49; Canelon Kakou) that contained a commercial potting soil mix (Saika-ichiban; Ibigawa Kogyo) and grown in the same glasshouse until the seedlings reached the three- to four-leaf stage.

### Preparation of the pathogen inoculum

*R. pseudosolanacearum* strain VT0801 (Marian *et al.*, 2018) was used as the pathogen in the present study. Strain VT0801 was pre-cultured on a casamino acid–peptone–glucose (CPG) agar plate (Kelman, 1954) at 30°C. After being incubated for 48 h, bacterial cells were transferred to CPG broth and cultured at 30°C with shaking at 200 rpm for 24 h. The culture broth was centrifuged at 9,900×*g* for 10‍ ‍min, and the precipitated cells were washed once with 10‍ ‍mM MgCl_2_·6H_2_O. Washed cells were resuspended in 10‍ ‍mM MgCl_2_·6H_2_O and adjusted to an OD_600_ of 0.1 (*ca.* 9×10^7^ colony-forming units [CFU] mL^–1^) or 1.0 (*ca.* 9×10^8^ CFU mL^–1^).

### Isolation of endophytic *Bacillus* sp.

We attempted to isolate endophytic *Bacillus* species with the potential of enhancing bacterial resistance in tomato plants. The procedure for the effective isolation of desirable target bacteria used in the present study consisted of the following steps: (i) enrichment of heat-tolerant bacteria in tomato plants, (ii) inoculation of tomato plants with the bacterial wilt pathogen, and (iii) isolation of heat-tolerant endophytic bacteria from asymptomatic tomato plants. The procedure is described in detail below.

Soil (100 g) was collected from pasture lands at the Minokamo Experimental Farm of Gifu University (Mikino, Minokamo city, Gifu Prefecture, Japan) and the tomato fields and eggplant fields at Gifu University (Yanagido, Gifu city, Gifu Prefecture, Japan). Soil was suspended in 500‍ ‍mL of SDW and pasteurized at 80°C for 20‍ ‍min to kill heat-sensitive Gram-negative bacteria and fungi. The aerial parts of three-leaf stage tomato seedlings grown on peat pellets were immersed in each pasteurized soil suspension supplemented with 0.01% (v/v) Silwet L-77 (Momentive Performance Materials Japan) for 1 h to enrich heat-tolerant *Bacillus* species in the seedlings. After immersion in the soil suspension, seedlings were placed in a glasshouse maintained at 28–30°C in natural sunlight. After 2 days, the roots protruding from peat pellets were cut off with sterile scissors, and the seedlings were drench-inoculated with 30‍ ‍mL of the VT0801 suspension (*ca.* 9×10^7^ CFU mL^–1^). These seedlings were placed in the same glasshouse and cultivated for 7 days. After cultivation, the leaves and stems were sampled from tomato seedlings that did not display any symptoms. Thereafter, leaf discs (5‍ ‍mm in diameter) and stem segments (*ca.* 1‍ ‍cm in length) were cut from the samples and surface-sterilized with 70% (v/v) ethanol for a few seconds, followed by 2% (v/v) sodium hypochlorite for 1‍ ‍min, and then rinsed five times in SDW. These samples were individually homogenized in 10‍ ‍mM MgCl_2_·6H_2_O using a sterile mortar and pestle. Subsequently, 1‍ ‍mL of the homogenate was transferred into a 1.5-mL tube and pasteurized in a hot water bath at 80°C for 10‍ ‍min to kill Gram-negative bacteria and fungi. After pasteurization, 100-μL aliquots of each homogenate were spread onto 9-cm plates of tryptic soy agar (TSA) medium (Difco) and incubated at 30°C for 2 days. Bacterial colonies appearing on the plates were transferred to freshly prepared TSA plates, purified by quadrant streaking, and stored in 10% (w/v) skim milk (Difco) supplemented with L-glutamic acid monosodium salt (16.5‍ ‍g L^–1^) at –80°C until used.

The surface sterilization procedure was validated through the following procedure: 100-μL aliquots of the last rinse were spread onto triplicate plates of TSA, and the absence of bacterial growth on the plates within 2 days of the incubation at 30°C indicated effective surface sterilization.

### Primary screening of the biocontrol strains

To screen biocontrol strains, we assessed the suppressive effects of bacterial strains against bacterial wilt on tomato seedlings grown on peat pellets using the following procedures: a loopful of the bacterial stock solution of each bacterial strain was inoculated into test tubes containing 10‍ ‍mL of tryptic soy broth (TSB) (Difco) and incubated at 30°C with shaking at 200 rpm for 24 h. Bacterial cells were harvested by centrifugation at 9,900×*g* for 10‍ ‍min. The precipitated cells were washed once with 10‍ ‍mM MgCl_2_·6H_2_O and resuspended in 10‍ ‍mM MgCl_2_·6H_2_O to an OD_600_ of 0.5. Cell suspensions were supplemented with 0.01% Silwet L-77 and used as the inoculum. One-week-old tomato seedlings grown on peat pellets (five seedlings per pellet) were treated by dipping their shoots into the cell suspension of each bacterial strain for 1 h. Control plants were subjected to the mock treatment (sterile 10‍ ‍mM MgCl_2_·6H_2_O supplemented with 0.01% Silwet L-77). After air-drying, the seedlings were placed in a glasshouse maintained at 28–30°C in natural sunlight. One day after treatment (dat), roots protruding from peat pellets were cut off with sterile scissors, drench-inoculated with 30‍ ‍mL of the VT0801 cell suspension (adjusted to *ca.* 9×10^6^ CFU mL^–1^), and then cultivated in the same glasshouse for 9 days.

In this experiment, we initially assessed the suppressive effects of all bacterial strains obtained from tomato plants against tomato bacterial wilt. As described later, we obtained 50 bacterial strains from tomato plants. These strains were divided into eight groups and their efficacy was assessed in eight separate trials (designated as initial trials). The symptoms of bacterial wilt were monitored daily on the basis of a disease scale that ranged from 0 to 2, where 0=no wilt symptoms (healthy), 1=partially wilted, 2=completely wilted. The disease severity and the area under disease progress curve (AUDPC) of each peat pellet were calculated using the following formulas: Disease severity=[(the number of plants in each disease scale × disease scale)/(total number of plants investigated × the highest disease scale)]×100%. AUDPC=Σ [0.5 (*x_i_*+*x_i–1_*)] (*t_i_*–*t_i–1_*), where *x_i_* and *x_i–1_* are the disease severity at time *t_i_* and *t_i–1_*; *t_i_* and *t_i–1_* are consecutive evaluation dates; and *t_i_*–*t_i–1_* is equal to 1. The reduction in AUDPC (%) was calculated from the following formula: Reduction in AUDPC (%)=[(mean of AUDPC_C_–mean of AUDPC_T_)/mean of AUDPC_C_]×100%, where AUDPC_C_ is the AUDPC value of the control treatment and AUDPC_T_ is the AUDPC value of the bacterial treatment.

The selected strains with an AUDPC reduction of ≥30% in the initial trials were then tested for their efficacy by repeating the same experiment three times (designated as the second trials). To compare the efficacy of the selected strains, wilt incidence data (the total number of wilted seedlings and total number of seedlings assessed) 9 days after the inoculation (dai) with the pathogen obtained from the initial and second trials (*i.e.*, data from four independent repeated trials) were subjected to a frequentist random-effects network meta-analysis using the R package “netmeta” (ver. 1.2-1) (Rücker, 2012; Schwarzer *et al.*, 2015).

### Pot experiment

The bacterial strains selected in the above primary screening were subjected to a pot experiment to compare the disease suppressive effects of spray treatments with these strains against tomato bacterial wilt. The stock solution of the selected bacterial strains was spread onto the surface of TSA plates (9‍ ‍cm in diameter) and pre-cultured at 30°C for 36 h. The bacterial cells were then harvested by washing with 2‍ ‍mL of SDW. The cell suspension of each strain was inoculated into 100‍ ‍mL of TSB and cultured at 30°C for 24 h with shaking at 200 rpm. The culture broth was centrifuged at 9,900×*g* for 10‍ ‍min and precipitated cells were washed once with 10‍ ‍mM MgCl_2_·6H_2_O. Bacterial cells were re-suspended in 10‍ ‍mM MgCl_2_·6H_2_O supplemented with 0.01% Silwet L-77 to OD_600_=1.0 (*ca.* 1×10^8^ CFU mL^–1^) and used as the inoculum.

Tomato seedlings (in the three- to four-leaf stage) grown on plastic trays as described above were transplanted into vinyl pots (9‍ ‍cm in diameter) comprising three layers: top, middle, and bottom. The top and bottom layers each contained 150‍ ‍g of commercial potting soil mix, and the middle layer contained 20‍ ‍g of river sands. The seedlings were treated by foliar spraying with the cell suspension of each bacterial strain until the leaves were evenly wet just before run-off, and then placed in a glasshouse maintained at 28–30°C in natural sunlight. The control plants received the mock treatment (10‍ ‍mM MgCl_2_·6H_2_O supplemented with 0.01% Silwet L-77) instead of the bacterial cell suspension. At 3 dat, control plants and those treated with bacterial strains were both drench-inoculated with 100‍ ‍mL of the VT0801-washed cell suspension (*ca.* 3×10^7^ CFU mL^–1^) to obtain a final concentration of *ca.* 1×10^7^ CFU g^–1^ soil. The incidence of wilt was recorded at 14 dai. Each treatment was applied to five or ten plants, and the experiment was repeated four times. Since the proportion of wilted plants in the control treatment markedly varied between repeated experiments, wilt incidence data (the total number of wilted plants at 14 dai and the total number of plants assessed) obtained from four independent repeated experiments were subjected to a frequentist network meta-analysis, as described above.

### Identification of selected strains

Two strains selected in the above primary screening experiment were identified based on the sequences of their 16S rRNA gene using the methods described by Nishioka *et al.* (2016). The genomic DNA of the strains was extracted using PrepMan Ultra sample preparation reagent (Applied Biosystems) in accordance with the manufacturer’s instructions. The 16S rRNA gene was amplified using the primers 27f and 1492r (Lane, 1991). Amplification conditions were as follows: one cycle of pre-denaturation at 94°C for 1‍ ‍min, followed by 25 cycles at 94°C for 1‍ ‍min, 55°C for 1‍ ‍min, and 72°C for 2‍ ‍min, with a final extension step at 72°C for 8‍ ‍min.

The amplified products were purified using the GenElute PCR Clean-Up Kit (Sigma). Cycle sequencing was performed with the Big Dye Terminator v3.1 Cycle Sequencing Kit (Applied Biosystems), 27f, 37f, 517r, and 1492r primers (Muyzer *et al.*, 1993), and an ABI PRISM 3100 Genetic Analyzer (Applied Biosystems). The cycle sequencing conditions used were as follows: 96°C for 1‍ ‍min (for initial denaturation), followed by 25 cycles at 96°C for 10‍ ‍s, 50°C for 5‍ ‍s, and 60°C for 4‍ ‍min. BLAST searches were performed for the sequences obtained to assess similarities with sequence data in GenBank. A phylogenetic tree was constructed by the neighbor-joining method using MEGA version 7.0.26 (Tamura *et al.*, 2013).

### Quantification of *R. pseudosolanacearum*

As described later, the spray treatment of tomato plants with strain G4L1 exerted significant biocontrol effects against bacterial wilt in the pot experiment. In this experiment, we examined the effects of the spray treatment with this strain on pathogen multiplication in tomato stems. Tomato plants that were transplanted into 9-cm pots were spray-treated with strain G4L1 and challenged with the pathogen, as described above. Control plants were mock-treated with 10‍ ‍mM MgCl_2_·6H_2_O supplemented with 0.01% Silwet L-77 and challenged with the pathogen. At 3, 7, and 14 dai, a 2-cm long stem was cut from the first leaf node (>1‍ ‍cm above the cotyledons) of symptomless plants. Three plants were cut at each time point. These stem samples were surface sterilized with 100% ethanol for a few seconds, flamed for a few sedonds, homogenized with nine volumes of SDW using a sterile mortar and pestle, and 10-fold serial dilutions were then prepared. Dilutions of the homogenate were spread in triplicate onto the surface of a modified semi-selective medium, South Africa (M-SMSA) (French *et al.*, 1995). Typical colonies of *R. pseudosolanacearum* that appeared elevated, fluidal, and with pink centers were counted after an incubation at 30°C for 3 days. The population was calculated as log CFU g^–1^ stem fresh weight. The experiment was repeated four times. Differences in the pathogen populations between the control and G4L1 treatments were analyzed by the Student’s *t*-test (*P*<0.05).

### Population dynamics of strain G4L1 on tomato leaves

Tomato seedlings (in the three- to four-leaf stage) grown on plastic trays, as described above, were transplanted into 9-cm vinyl pots containing a commercial potting soil mix. The seedlings were then spray-treated with the washed cell suspension of strain G4L1 (*ca.* 1×10^8^ CFU mL^–1^) supplemented with 0.01% Silwet L-77 until run-off, placed in the glasshouse, and maintained at 28–30°C in natural sunlight. Control plants received the mock treatment (10‍ ‍mM MgCl_2_·6H_2_O supplemented with 0.01% Silwet L-77). The population of strain G4L1 on/in the first to third leaflets and stems was assessed at 1, 7, 14, 21, and 28 dat. Samples were obtained from three plants at each time point. Three leaves were excised from each leaflet. A 2-cm-long stem was excised from the second leaf node. These leaf and stem samples were homogenized and then serially diluted. Dilutions of homogenates were spread in triplicate onto the surface of TSA plates. As shown in Supplementary Fig. S4, strain G4L1 grew very rapidly and formed unique colonies that were distinguishable from other bacterial colonies within 24 h of incubation. Therefore, the number of bacterial colonies with characteristics specific to strain G4L1 was counted after cultivation at 30°C for 24 h and the population of this strain was calculated as log CFU g^–1^ leaf fresh weight or log CFU g^–1^ stem fresh weight. The experiment was repeated three times.

### Analysis of tomato defense‑related gene expression using quantitative real-time PCR (qRT-PCR)

This experiment comprised three treatments: 1) control (mock-treated and uninoculated with the pathogen); 2) inoculated control (mock-treated and inoculated with the pathogen); and 3) G4L1+ pathogen (treated with strain G4L1 and inoculated with the pathogen). The G4L1 treatment and pathogen inoculation were performed as described for the pot experiment. These seedlings were placed in a growth chamber with a controlled environment (Plant Growth Chamber CLE-405; TOMY SEIKO) at 28°C with a 14-h light/10-h dark cycle.

At 3 dai, the middle part of the stem (*ca.* 0.20 g, approximately 1‍ ‍cm above the cotyledons) and the main root (*ca.* 0.10 g) were sampled from tomato plants to analyze the expression of the *PR-1a* (pathogenesis-related protein 1a) and *GluA* (acidic β-1,3-glucanase), *GluB* (basic β-1,3-glucanase) and *OLP* (osmotin-like protein), *LoxD* (lipoxygenase D), and *Le4* (late embryogenesis abundant protein) genes, which are related to the salicylic acid (SA), ethylene (ET), jasmonic acid (JA), and abscisic acid (ABA) signaling pathways, respectively. All samples taken from plants were immediately frozen in liquid nitrogen and stored at –80°C until RNA extraction.

Regarding RNA extraction, samples were transferred to Lysing Matrix Tubes and powdered under dry-ice cooling using FastPrep-24^TM^ 5G (MP Biomedicals). RNA was extracted as described previously (Marian *et al.*, 2019). After RNA concentrations were measured using a NanoVue Plus Spectrophotometer (GE Healthcare Life Sciences), 400‍ ‍ng of total RNA was used to synthesize first-strand cDNA and as templates for qRT-PCR.

The qRT-PCR reaction analysis used a total volume of 10‍ ‍μL containing 3‍ ‍μL of RNase-free water, 5‍ ‍μL of 2 SYBR Premix (Tli RNaseH Plus; Takara Bio), 1‍ ‍μL of a cDNA template, and 0.5‍ ‍μL of 10‍ ‍μM of each forward and reverse gene-specific primer with a LightCycler Nano Instrument (Roche Diagnostics) in a two-step reaction (an initial denaturation step, a three-step amplification profile), as described previously (Marian *et al.*, 2019). The housekeeping gene β-tubulin was used for normalization. The expression of the target genes in different samples was calculated using the formula 2^–ΔΔCT^ (Livak and Schmittgen, 2001) and presented as a value relative to that of the control treatment. This experiment was conducted once with three biological replicates for each treatment and two or three technical repetitions for each replicate. Differences in gene expression among treatments were analyzed by Tukey’s test (*P*<0.05).

### Statistical analysis

All statistical analyses were performed with EZR version 1.41 (Saitama Medical Center, at http://www.jichi.ac.jp/saitama-sct/SaitamaHP.files/statmedEN.html), which is a graphical user interface for R (The R Foundation for Statistical Computing, version 3.6.1).

### Nucleotide sequence accession numbers

The nucleotide sequences of the 16S rRNA genes were deposited in the GenBank database under accession numbers MT543223–MT543224.

## Results

### Isolation of endophytic bacteria

To obtain endophytic *Bacillus* species with the potential to induce bacterial wilt resistance in tomato plants, we isolated heat-tolerant bacteria from the surface-sterilized tissues of asymptomatic tomato seedlings inoculated with the bacterial wilt pathogen. Several bacterial colonies appeared on the surface of TSA plates 2 days after spreading the dilution of tissue homogenates pasteurized at 80°C. These bacteria were presumed to be endophytic because no bacterial colonies appeared on TSA plates upon the incubation of the last washing solution. These bacteria were purified using quadrant streaking methods. Accordingly, 50 endophytic bacteria were successfully isolated. Among them, 28 strains were recovered from leaves, whereas the remaining 22 were recovered from the stems (Table 1).

### Primary screening

**Fig. 1.**
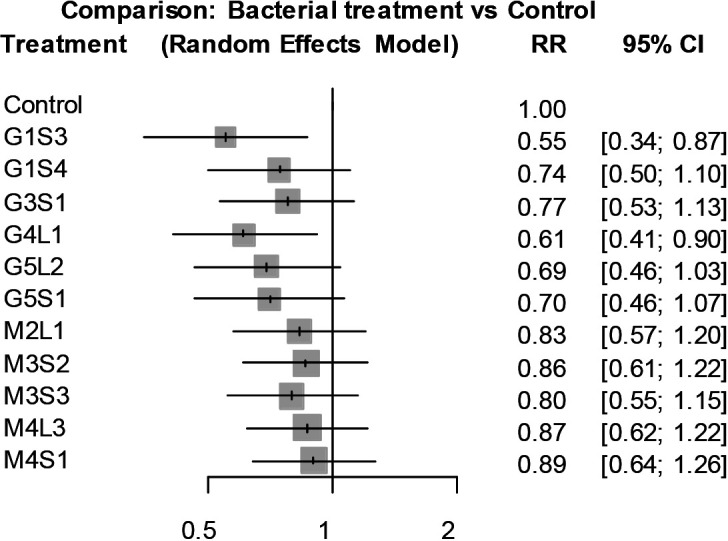
Forest plot of the comparison between bacterial treatments and control for the incidence of tomato bacterial wilt in the primary screening experiment. Wilt incidence data (the number of wilted seedlings at 9 dai) obtained from four independent repeated trials were analyzed by a frequentist network meta-analysis. The grey boxes indicate the relative risk (RR) for individual treatments and the horizontal bars indicate the corresponding 95% confidence interval (95% CI).

Using tomato seedlings grown on peat pellets, the effects of the shoot-dipping treatment on bacterial wilt was assessed. In preliminary trials (*i.e.*, initial trials), all 50 bacterial strains were screened for their wilt suppressive effects. The results obtained showed that 25 out of 50 strains suppressed the progress of bacterial wilt to a greater or lesser extent (Fig. S1 and S2). Among the 25 strains, 11 reduced the AUDPC value by more than 30% from that by the mock control (Fig. S2). Therefore, the wilt suppressive effects of the 11 strains (G1S3, G1S4, G3S1, G4L1, G5L2, G5S1, M2L1, M3S2, M3S3, M4L3, and M4S1) were examined in more detail in the efficacy confirmation trial (*i.e.*, the second trial). Although these strains suppressed disease progression in the preliminary trials, eight failed to reduce the number of wilted seedlings at 9 dai in one or two of the three repeated experiments. In contrast, strains G1S3 and G4L1 consistently reduced the incidence of wilt to lower than that by the mock control throughout repeated experiments. In the results of a network meta-analysis of wilt incidence data obtained from the preliminary and efficacy confirmation trials, the relative risk (RR) values of the G1S3 and G4L1 treatments were 0.55 (95% confidence interval: 0.34–0.87) and 0.61 (95% confidence interval: 0.41–0.90), respectively, indicating that the incidence of bacterial wilt was significantly suppressed by the shoot-dipping treatment with these two strains (Fig. 1). The RR values of 0.55 and 0.61 indicated that the G1S3 and G4L1 treatments reduced the incidence of bacterial wilt to 55 and 61% of that of the control treatment, respectively.

### Identification of selected strains

**Fig. 2.**
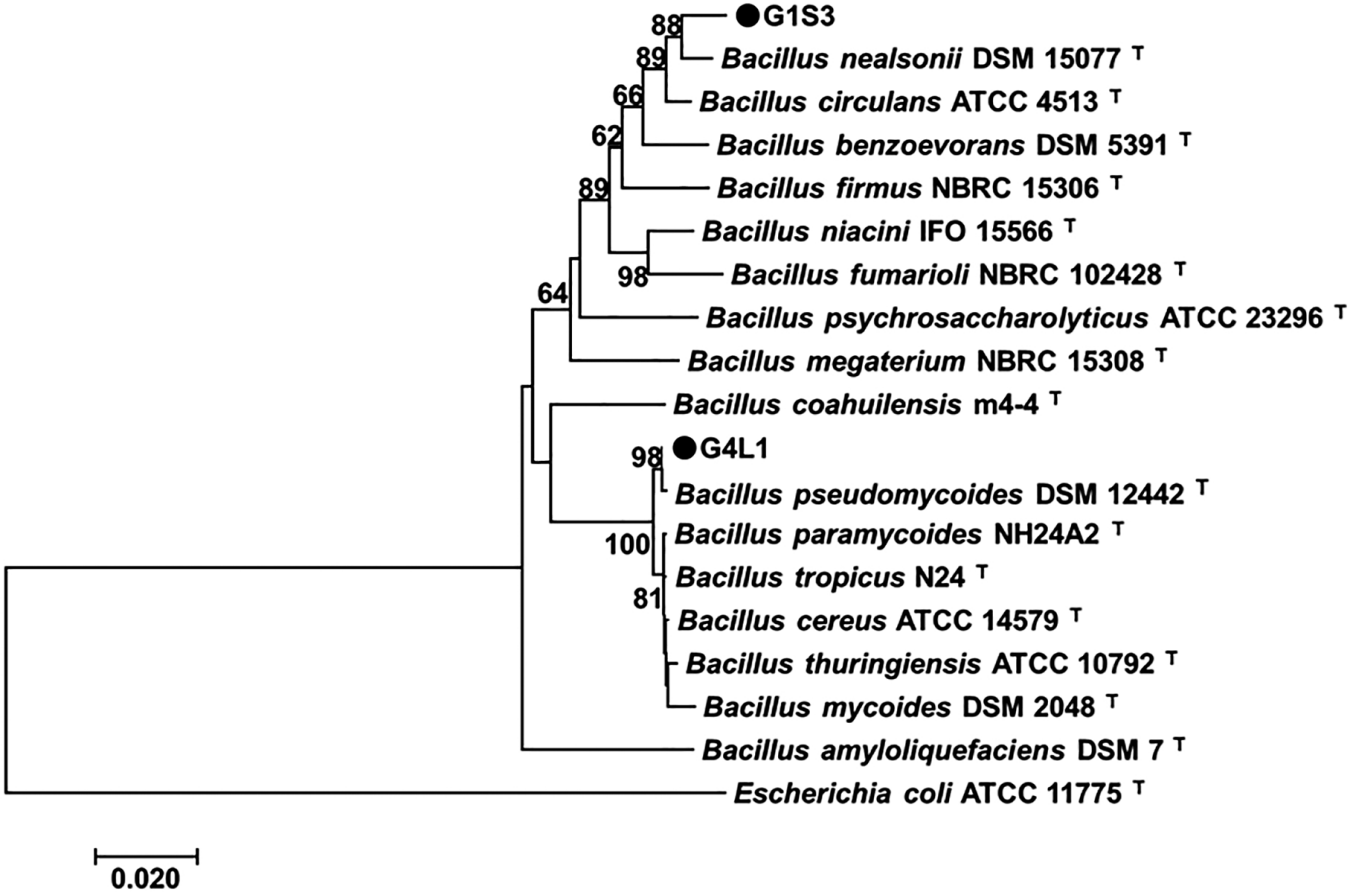
Phylogenetic tree derived from 16S rRNA gene sequence data of two selected strains (G1S3 and G4L1) and their relatives. The tree was generated by the neighbor-joining method, and genetic distances were calculated by the Kimura 2-parameter method using MEGA ver. 7.0.27 (Tamura *et al.*, 2013). *Escherichia coli* (ATCC 11775) was used as an outgroup to root the tree. Numbers at nodes are percentage bootstrap values (only bootstrap values higher than 60% are shown from 1,000 replications). The scale bar represents 0.02 nucleotide substitutions per nucleotide position.

The analysis of the full-length 16S rRNA sequences of these two strains indicated that G1S3 (approximately 1,222‍ ‍bp, accession no. MT543223) shared 98% identity with that of *Bacillus nealsonii* strain DSM 15077^T^ (accession no. EU656111), whereas strain G4L1 (approximately 1,207 bp, accession no. MT543224) showed 99% identity with that of *B. pseudomycoides* strain DMS 12242^T^ (accession no. Mn543762). To clarify the phylogenetic position of both strains, a phylogenetic tree was constructed based on the 16S rRNA gene sequences of both strains and their closest relative type strains (Fig. 2). Strains G1S3 and G4L1 showed clear distinctions from the known type strains of *B. nealsonii* and *B. pseudomycoides*, respectively. Therefore, we identified them as *Bacillus* spp.

### Pot experiment

**Fig. 3.**
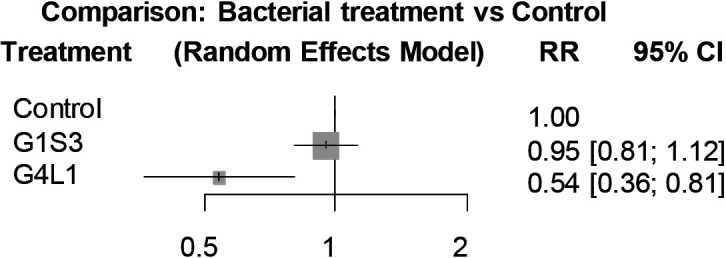
Forest plot of a network meta-analysis comparing bacterial treatments and the control treatment for the incidence of tomato bacterial wilt in the pot experiment. Wilt incidence data (the number of wilted seedlings at 14 dai) obtained from four independent repeated trials were analyzed by a frequentist network meta-analysis. The grey boxes indicate the relative risk (RR) for individual treatments and the horizontal bars indicate the corresponding 95% confidence interval (95% CI).

The biocontrol efficacy of the foliar spray treatment with strains G1S3 and G4L1 was evaluated in four independent pot trials that were performed under glasshouse conditions. In the control treatment, signs of wilting started within 5 to 8 dai on tomato plants, and disease incidence reached 60–100% by 14 dai (Table 2 and Fig. S3A). Tomato plants treated with G1S3 showed a slightly lower disease incidence (40–90%) than that of the control treatment in three out of four trials (Table 2 and Fig. S3B). In contrast, the G4L1 treatment consistently suppressed the development of bacterial wilt more than the control and reduced disease incidence by 50% or less throughout the four repeated trials (Table 2 and Fig. S3C). As shown in Fig. 3, a network meta-analysis of data from four trials showed that the RR values of the G4L1 and G1S3 treatments were 0.54 (95% confidence interval: 0.36–0.81) and 0.95 (95% confidence interval: 0.81–1.12), respectively (Fig. 3), indicating that foliar spraying with G4L1 exhibited a significant control effect on tomato bacterial wilt, whereas the G1S3 treatment did not. Based on this result, strain G4L1 was selected as the final biocontrol candidate and subjected to the following experiments.

### Quantification of *R. pseudosolanacearum*

**Fig. 4.**
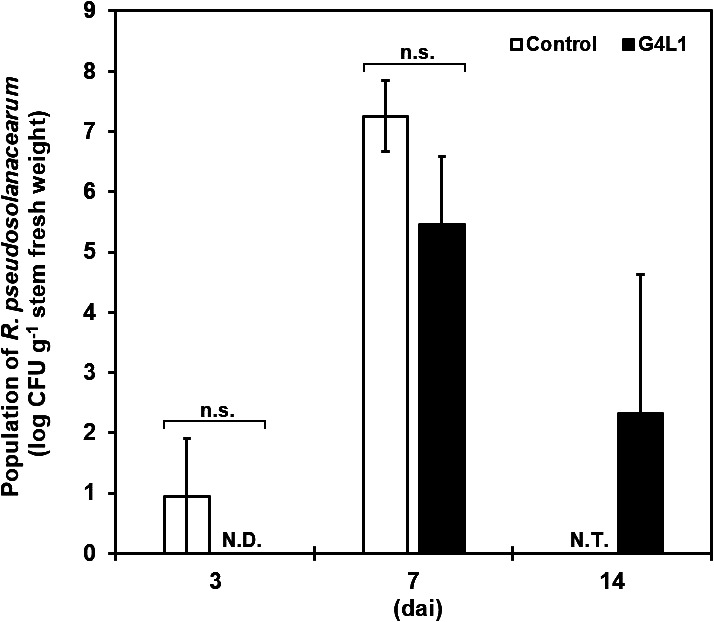
Population dynamics of *Ralstonia pseudosolanacearum* in the stem of tomato plants spray-treated with the biocontrol *Bacillus* strain G4L1. Bars represent the mean±standard error. N.D., not detected. N.T., not tested; n.s., not significant (*P*<0.05 using the Student’s *t*-test).

The *R. pseudosolanacearum* population in the stem tissue of symptomless tomato plants treated and untreated with strain G4L1 was assessed at 3, 7, and 14 dai. At 14 dai, all control plants were completely wilted; thus, the pathogen population in these plants was not investigated. In the control treatment, the pathogen population was detected in the stem (*ca.* 1.0 log CFU g^–1^ stem fresh weight) at 3 dai (Fig. 4). At 7 dai, the pathogen population reached *ca.* 7.3 log CFU g^–1^ stem fresh weight. In contrast, the pathogen was not detected in the stems of G4L1-treated plants at 3 dai. At 7 dai, the pathogen population in G4L1-treated plants (5.5 log CFU g^–1^ stem fresh weight) was always lower, but not significantly lower (*P*=0.205), than that in control plants throughout the four repeated trials. The pathogen population in G4L1-treated plants decreased to *ca.* 2.3 log CFU g^–1^ stem fresh weight by 14 dai.

### Population dynamics of G4L1 after the foliar spray treatment

**Fig. 5.**
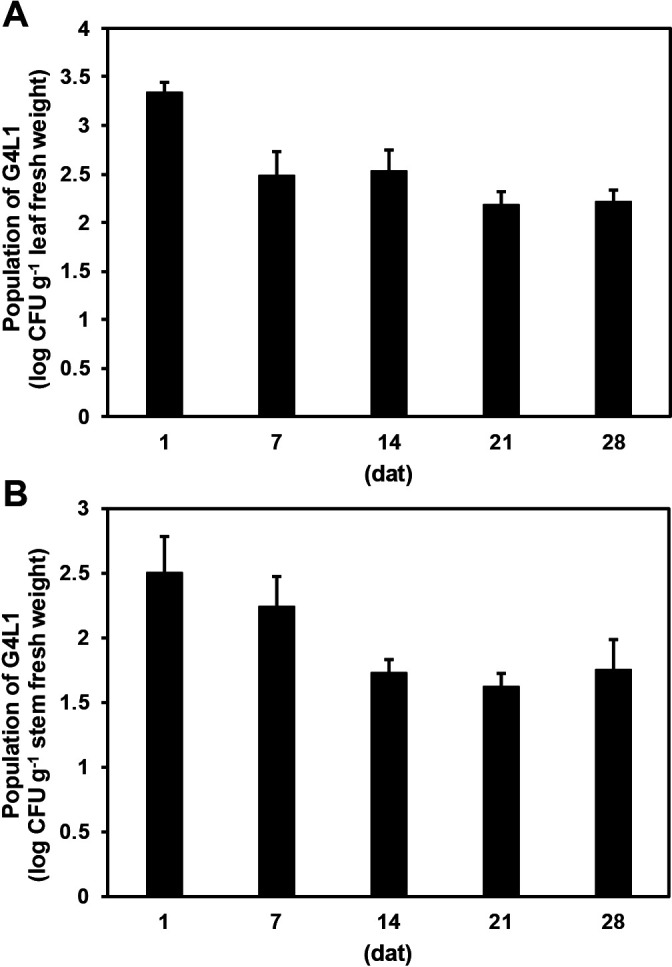
Population of dynamics of the biocontrol *Bacillus* strain G4L1 on leaves (A) and stems (B) of tomato plants. Bars represent the mean±standard error.

The population of strain G4L1 on the foliar tissues of tomato plants was investigated at 1, 7, 14, 21, and 28 dat under glasshouse conditions. G4L1 was successfully recovered from both the leaves and stems of spray-treated tomato plants during growth in the glasshouse (Fig. 5). At 1 dat, the strain was detected at *ca.* 3.3 log CFU g^–1^ fresh weight and *ca.* 2.5 log CFU g^–1^ fresh weight in the leaves and stems, respectively (Fig. 5). In the leaves, the population of strain G4L1 slightly decreased to 2.5 log CFU g^–1^ fresh weight by 7 dat and thereafter was stably maintained until 28 dat (Fig. 5A). In contrast, the G4L1 population in the stems gradually declined by *ca.* 1.7 log CFU g^–1^ fresh weight during the first 14 days and then stabilized over the following 2‍ ‍weeks (Fig. 5B). G4L1-like colonies were not detected in either the leaves or stems of mock-treated control tomato plants.

### Expression of defense-related genes in tomato plants

**Fig. 6.**
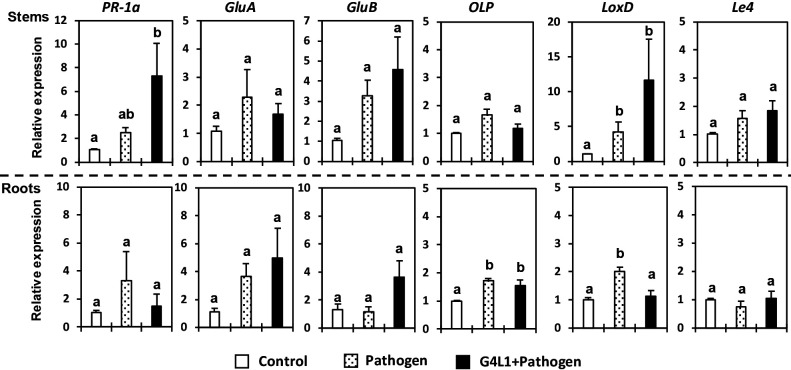
Expression of defense-related genes in main roots and stems of G4L1-treated or mock-treated tomato plants inoculated without or with *Ralstonia pseudosolanacearum* 3 days after the pathogen inoculation. The housekeeping gene *β-tubulin* was used for normalization. The expression levels of the target genes in different samples were calculated using the formula 2^–ΔΔCT^ (Livak and Schmittgen, 2001), given as a value relative to mock-treated control plants (not inoculated with the pathogen). Bars represent the mean±standard error of three biological replicates per treatment with two or three technical repetitions for each sample. Different letters indicate significant differences between treatments according to Tukey’s test at *P*<0.05.

We investigated the effects of the foliar spray treatment with G4L1 on the expression of the six defense-related genes in the stems and main roots of tomato plants at 3 dai (6 dat). The results of the qRT-PCR analysis are shown in Fig. 6.

In the stems of mock-treated plants, the expression of SA-responsive marker genes *PR-1a* and *GluA*, ET-responsive *GluB*, and JA-responsive *LoxD* was slightly up-regulated by the inoculation with the pathogen. In contrast, the expression of SA-responsive *PR-1a* and JA-responsive *LoxD* was significantly stronger in the stems of G4L1-treated plants upon the pathogen inoculation than in those of uninoculated control plants. The expression of *GluB* was also slightly up-regulated in G4L1-treated pathogen-inoculated plants, but was similar to that in mock-treated pathogen-inoculated plants. Neither of the treatments induced the ABA-responsive *Le4* gene.

In the roots of mock-treated plants, ET-responsive *OLP* and JA-responsive *LoxD* were significantly up-regulated by the pathogen inoculation. In addition, the expression of SA-responsive *PR-1a* and *GluA* was also slightly induced in the roots of mock-treated pathogen-inoculated plants. In the roots of G4L1-treated plants, the expression of *OLP* was significantly up-regulated; however, its expression level was similar to that in the roots of mock-treated pathogen-inoculated plants. The expression of *GluA* was also slightly up-regulated, to a similar level as that in the roots of mock-treated pathogen-inoculated plants. The expression level of *GluB* was slightly higher (*P*=0.074) in the roots of G4L1-treated plants than in those of uninoculated control plants. ABA-responsive *Le4* was not induced following either treatment.

## Discussion

The aim of the present study was to screen ISR-inducing endophytic *Bacillus* isolates capable of suppressing tomato bacterial wilt by foliar spray application. We isolated 50 heat-tolerant bacteria as presumptive *Bacillus* species from the surface-sterilized foliar tissues of symptomless tomato plants that had been pre-inoculated with the bacterial wilt pathogen (Table 1).

Many different approaches have been developed to screen potential BCAs against plant pathogens. In the majority of biocontrol studies, the direct antagonistic effects of microbial isolates on pathogens (such as antibiosis, parasitism, and competition) have been tested in a preliminary screening using *in vitro* methods, such as dual culture assays and lytic enzyme assays (Pliego *et al.*, 2011). In contrast, only a few studies investigated the isolates of BCAs that exert biocontrol effects via the induction of ISR (Raymaekers *et al.*, 2020). In the present study, we applied our 50 bacterial strains to the foliar tissues of tomato seedlings to test their ability to induce ISR against root infection with the bacterial wilt pathogen. Accordingly, strain G4L1 was selected as a potential BCA because of its consistent ability to suppress tomato bacterial wilt across primary screening trials and pot experiments (Fig. 1 and 3). Since strain G4L1 was applied to foliar tissues only, its protective effects were caused by a plant-mediated phenomenon and, thus, must be ascribed to ISR. The foliar spraying of strain G4L1 significantly suppressed the incidence of tomato bacterial wilt for up to 2‍ ‍weeks under very high pathogen pressure (approximately 10^7^ CFU g^–1^ soil) in pot experiments (Fig. 3 and S3C). Many studies have reported the biocontrol of bacterial wilt by the application of antagonistic BCAs into the plant rhizosphere (Ramesh and Phadke, 2012; Wei *et al.*, 2013; Xue *et al.*, 2013; Chen *et al.*, 2014; Marian *et al.*, 2018). Moreover, the suppressive effects of leaf infiltration with ISR-inducing fluorescent pseudomonads against the bacterial wilt of Eucalyptus have been reported (Ran *et al.*, 2005). However, to the best of our knowledge, this is the first study to report the biocontrol of bacterial wilt disease by the foliar spray application of BCAs. Plant activators, such as benzothiadiazole S-methyl ester, acibenzolar-S-methyl, and validamycin A, occasionally cause a reduction in plant growth or phytotoxic symptoms (Godard *et al.*, 1999; Ishikawa *et al.*, 2007; Kunwar *et al.*, 2017). In contrast, strain G4L1 did not exert any adverse effects on tomato plants (data not shown). These results indicate that strain G4L1 has potential as a novel biological plant activator for the control of tomato bacterial wilt.

Phylogenetic analyses based on 16S rRNA sequences showed that strain G4L1 was the species most closely related to *B. pseudomycoides* (Fig. 2). The biocontrol potential of *B. pseudomycoides* against bacterial wilt disease has recently been reported by two research groups; Hassan *et al.* (2017) demonstrated the biocontrol activity of rhizospheric *B. pseudomycoides* against bacterial wilt in tomato plants, and Yanti *et al.* (2018) identified endophytic *B. pseudomycoides* from chili pepper roots as an effective BCA against the bacterial wilt of chili pepper. Although strain G4L1 was phylogenetically close to *B. pseudomycoides*, this strain may be distinct from this species because its colony morphology is different from that of *B. pseudomycoides*. Strain G4L1 produced round to irregular colonies with slightly undulating margins (Fig. S4), whereas *B. pseudomycoides* formed rhizoidal colonies (Nakamura, 1998). The species *B. pseudomycoides* is included in the *Bacillus cereus sensu lato* group, which comprises numerous closely related species, such as *Bacillus cereus*, *Bacillus thuringiensis*, *Bacillus mycoides*, *Bacillus weihenstephanensis*, and *Bacillus anthracis* (Fayad *et al.*, 2019). The 16S rRNA gene sequence may not be sufficient to differentiate the species within this group because of its high conservation (Okinaka and Keim, 2016; Liu *et al.*, 2017). Therefore, more detailed molecular characterization, using whole-genome sequencing, will be necessary to accurately assign strain G4L1 to a particular species.

The pathogen population in the stem tissues of G4L1-treated plants was markedly smaller than in those of mock-treated control plants for up to at least 7 dai (Fig. 4). Furthermore, even at 14 dai, the pathogen population in this treatment (*ca.* 2.3 log CFU g^–1^, equal to *ca.* 3.3×10 CFU cm^–1^) remained below the threshold population (1.18×10^8^ CFU cm^–1^) for the onset of wilt symptoms (Huang and Allen, 2000). This reduced population may have been due to the defense responses elicited by strain G4L1 because the suppression of pathogen growth in the stem tissue of G4L1-treated plants was accompanied by the induction of defense-related gene expression in stem tissues. The results from the qRT-PCR analysis revealed that the expression of the SA-dependent gene *PR-1a* and JA-dependent gene *LoxD* was markedly stronger in the stem tissues of G4L1-treated plants upon the pathogen inoculation than in those of pathogen-inoculated and uninoculated control plants (Fig. 6). Moreover, the expression of the ET-dependent gene *GluB* was slightly up-regulated in the roots of G4L1-treated plants upon the pathogen inoculation, whereas that in pathogen-inoculated control plants was not induced (Fig. 6). These results suggested that strain G4L1 activated defense responses by stimulating the SA-, JA-, and ET-signaling pathways and contributed to the suppression of pathogen multiplication in stem tissues, and possibly root infection by the pathogen. SA-mediated defenses generally inhibit biotrophic/hemibiotrophic pathogens, including *R. (pseudo)solanacearum*, whereas JA-/ET-mediated defenses generally act against necrotrophic pathogens (Lemarié *et al.*, 2015; Blake *et al.*, 2016). Many studies demonstrated the involvement of SA-dependent resistance in the biocontrol of bacterial wilt disease (Ran *et al.*, 2005; Feng *et al.*, 2012; Hyakumachi *et al.*, 2013; Takahashi *et al.*, 2014). However, the contribution of the JA/ET-signaling pathway to BCA-mediated resistance against bacterial wilt has also been reported (Hase *et al.*, 2008; Nakahara *et al.*, 2016). Moreover, several recent studies indicated that BCAs trigger resistance against bacterial wilt via the coactivation of the SA- and JA/ET-signaling pathways (Tan *et al.*, 2013; Li *et al.*, 2017). The signaling pathways involved in the elicitation of BCA-mediated ISR may be activated in response to the recognition of conserved microbe-specific molecules, such as lipopolysaccharides and chitin, or other elicitor compounds, including siderophores and antibiotics (Köhl *et al.*, 2019). Several antimicrobial cyclic lipopeptides (CLPs) from biocontrol *Bacillus* strains, such as iturin A, fengycin, and surfactin, have been identified as elicitors that induce SA- and/or JA-dependent resistance in various plants, including tomato (Ongena *et al.*, 2007; Kawagoe *et al.*, 2015; Tunsagool *et al.*, 2019). Furthermore, Tahir *et al.* (2017) reported that the volatile organic compounds (VOCs) produced by *Bacillus amyloliquefaciens* FZB42 and *Bacillus artrophaeus* LSSC22 induced SA-dependent defense responses against bacterial wilt pathogens in tobacco plants. In the present study, we did not examine the ISR-inducing effects of the compounds produced by strain G4L1. Thus, it currently remains unclear whether our strain has the capacity to synthesize similar CLPs or VOCs with elicitor activity. Further studies are needed to elucidate the signal transduction pathways and identify the elicitor molecules involved in the elicitation of bacterial wilt resistance by strain G4L1.

The results of the re-isolation experiment demonstrated that strain G4L1 efficiently colonized the leaves and stems of tomato plants for 4‍ ‍weeks after spray application (Fig. 5). Although we did not perform quantitative comparisons, the growth speed of G4L1 on agar medium was the fastest among the bacterial strains used in the present study. This fast-growing ability of strain G4L1 may contribute to the efficient colonization of tomato plants by this strain. The efficiency of BCAs strongly depends on colonization and survival at the target sites (Lee and Ryu, 2016; Mendis *et al.*, 2018; Gamez *et al.*, 2019). Therefore, the capacity of strain G4L1 to colonize the foliar tissues of tomato plants after foliar spray application is one of the reasons for its stable and superior biocontrol efficacy. This result also suggests that this strain protects tomato plants for up to 4‍ ‍weeks; however, the durability of the biocontrol effect of strain G4L1 currently remains unclear. In contrast, strain G1S3, which was one of the two candidate strains selected in the primary screening, did not suppress bacterial wilt in the pot experiments. Although we did not investigate the colonization ability of G1S3, this strain may not be able to efficiently colonize tomato shoots after spray application, and this may be because of its slower growth rate or other unknown factors; therefore, it failed to exert a biocontrol effect in the pot experiments. Strain G4L1 was originally isolated as a presumptive endophyte from the surface-sterilized leaf tissue of tomato plants. However, in the present study, we did not elucidate the mechanisms by which this strain colonized tomato plants. Therefore, further studies are needed to clarify the colonization pattern of strain G4L1 in tomato plants.

In conclusion, the present results demonstrated that the foliar spray application of *Bacillus* sp. G4L1 effectively suppressed tomato bacterial wilt, and thus, may contribute to the development of a novel biocontrol product that controls bacterial wilt disease. Studies are underway to elucidate the mechanisms of bacterial wilt resistance induced by strain G4L1. Further studies are needed to verify the efficacy of this strain in tomato under field conditions.

## Citation

Fu, H.-Z., Marian, M., Enomoto, T., Hieno, A., Ina, H., Suga, H., and Shimizu, M. (2020) Biocontrol of Tomato Bacterial Wilt by Foliar Spray Application of a Novel Strain of Endophytic *Bacillus* sp.. *Microbes Environ ***35**: ME20078.

https://doi.org/10.1264/jsme2.ME20078

## Supplementary Material

Supplementary Material

## Figures and Tables

**Table 1.** The number of bacterial strains obtained from surface-sterilized tissues of tomato plants

Plants	Tissue	No. of strains	Strain code
Tomato seedlings pretreated with a suspension of tomato field soil	Leaf	9	P#L#
	Stem	5	P#S#
Tomato seedlings pretreated with a suspension of eggplant field soil	Leaf	8	G#L#
	Stem	10	G#S#
Tomato seedlings pretreated with a suspension of pastureland soil	Leaf	11	M#L#
	Stem	7	M#S#

**Table 2.** Effects of the spray application of two selected bacterial strains on the disease incidence of tomato bacterial wilt in a pot experiment.

Treatment	Disease incidence (%)^a^			
	Trial 1 (*n*=5)^b^	Trial 2 (*n*=5)	Trial 3 (*n*=10)	Trial 4 (*n*=10)
Control	80	60	70	100
G1S3	100	40	60	90
G4L1	40	40	40	50

^a^ Disease incidence (%)=[Σ (no. of plants with wilt symptoms)/(total no. of plants assessed)]×100%. ^b^ The experiment was repeated four times (trial 1–4). “*n*” is the number of plants used for each treatment.
