# Supplementary material for: Biocontrol of Tomato Bacterial Wilt by Foliar Spray Application of a Novel Strain of Endophytic Bacillus sp
Source: Microbes Environ. 2020 Oct 2;35(4):ME20078. doi: 10.1264/jsme2.ME20078 (PMC7734409; doi:10.1264/jsme2.ME20078)
Supplement: Supplementary file 1 — Supplementary Material [file 35_20078_s1.pdf]

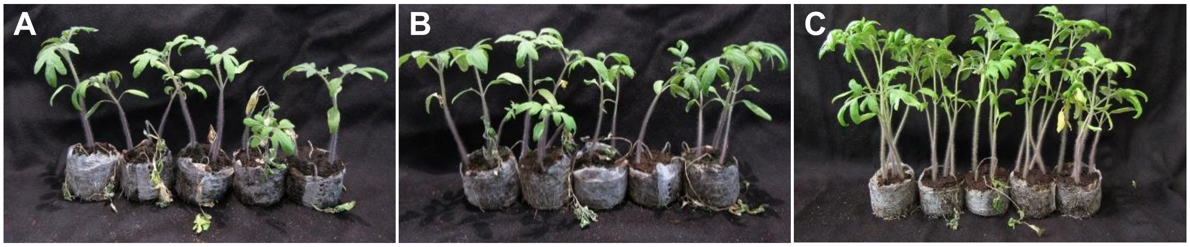

Fig. S1. Examples of wilt suppressive effects provided by shoot-dipping treatment with bacterial strains in the primary screening trial. Mock-treated seedlings showing severe wilt symptoms (A), Bacteria-treated seedlings showing mild (B), and lesser symptoms (C). Photos were taken at 9 dai.

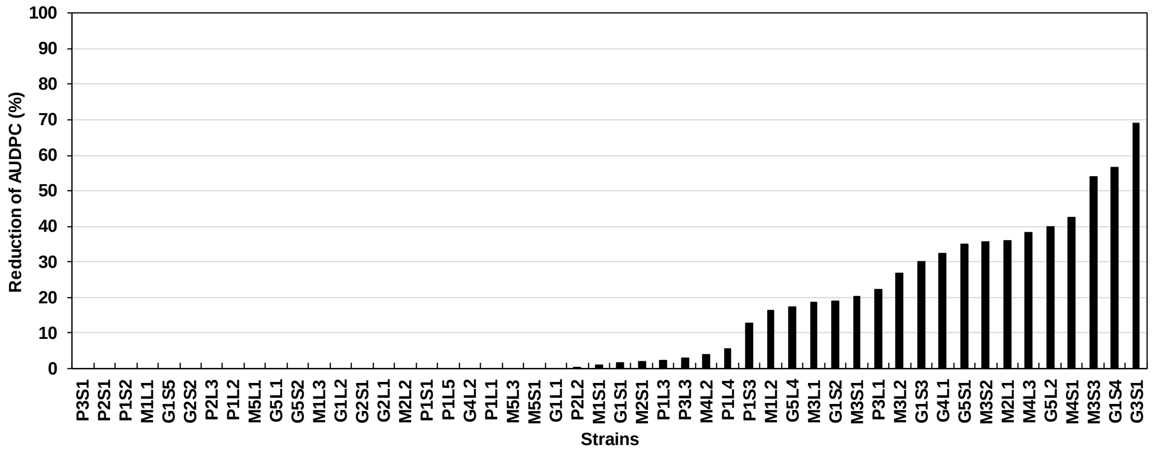

Fig. S2. Suppressive effect of bacterial strains against tomato bacterial wilt in the initial trials of primary screening experiment. Disease severity = [(the number of plants in each disease scale × disease scale) / (total number of plants investigated × the highest disease scale)] × 100%. Reduction of AUDPC (%) was calculated based on AUDPC data by the following formula: Reduction of AUDPC (%) = [(mean of $AUDPC_C$ – mean of $AUDPC_T$) / mean of $AUDPC_C$] ×100%, where $AUDPC_C$ is the AUDPC value of control treatment and $AUDPC_T$ is the AUDPC value of bacterial treatment. Negative values were converted to zero.

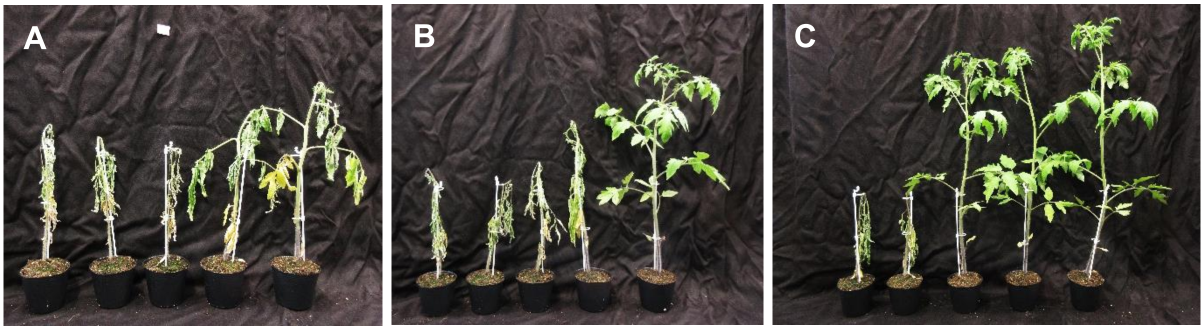

Fig. S3. Suppressive effect of spray treatment with strains G1S3 and G4L1 against tomato bacterial wilt in the pot experiment. (A) Control, (B) G1S3 treatment, (C) G4L1 treatment. Photos were taken at 14 dai.

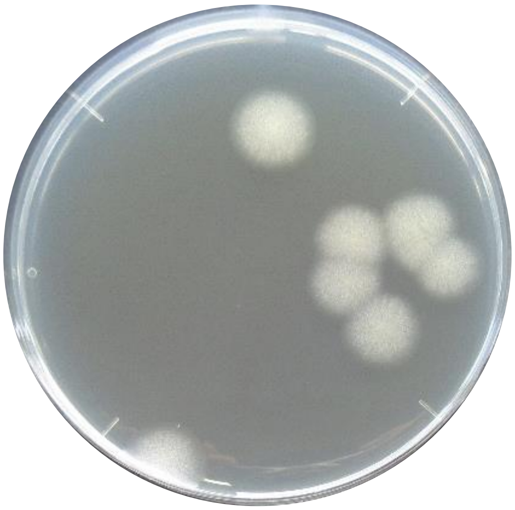

Fig. S4. Colonies of strain G4L1 on the TSA plate. Photo was taken at 24 h after the inoculation of the TSA plate with a diluted cell suspension of strain G4L1. Strain G4L1 grows quickly and produces large flat colonies having light yellow to light greenish-yellow color within 24 h of incubation.
